# Knowledge and attitudes toward repetitive transcranial magnetic stimulation (rTMS) as a treatment for postpartum and peripartum depression

**DOI:** 10.1192/j.eurpsy.2022.1910

**Published:** 2022-09-01

**Authors:** H. Al-Shamali, N. Zinchuk, R. Yan, M. Jackson, M. Morrissette, A. Greenshaw, Y. Zhang

**Affiliations:** 1 University of Alberta, Psychiatry, Edmonton, Canada; 2 University of Alberta, Nursing, Edmonton, Canada

**Keywords:** peripartum depression, repetitive transcranial magnetic stimulation, knowledge, postpartum depression

## Abstract

**Introduction:**

Postpartum and peripartum depression are debilitating disorders that impact the mother and their ability to care for their children’s emotional, social, and physical needs. Current treatments include psychotherapy, pharmacotherapy, and electroconvulsive therapy. These treatments are moderately effective or come with side effects that can negatively impact mother and child. As a result, many mothers view some treatments as unacceptable while pregnant or breastfeeding. Over the last decade, repetitive transcranial magnetic stimulation (rTMS) has shown promise as an effective and safe treatment option for postpartum and peripartum depression. However, little is known regarding people’s knowledge and attitudes towards this emerging technology, with no research assessing this in Canada.

**Objectives:**

We aim to identify gaps in knowledge and to assess attitudes toward rTMS as a treatment for postpartum and peripartum depression in mental health professionals, patients, and the general public living in Canada.

**Methods:**

A mixed methods study design will be employed. The qualitative portion will consist of individual semi-structured interviews. An inductive thematic analysis will be completed. The quantitative portion will consist of an anonymous, self-administered survey shared through REDCap. Focus groups with rTMS experts will be conducted to inform survey creation.

**Results:**

No resulst at this time.

**Conclusions:**

Understanding gaps in knowledge and attitudes toward rTMS is the first step toward ensuring that everyone is well informed and able to access safe and effective treatments. With limited treatment options available to a postpartum and/or peripartum depression patients being well informed on all treatments is crucial towards accessing treatments that best suit their needs.

**Disclosure:**

No significant relationships.

